# Pathophysiology of Spontaneous Coronary Artery Dissection Determines Anticoagulation Strategy

**DOI:** 10.7759/cureus.17437

**Published:** 2021-08-25

**Authors:** Philip Houck

**Affiliations:** 1 Medicine/Cardiology, Texas A&M Health Sciences Center, Temple, USA; 2 Medicine/Cardiology, Baylor Scott & White Health, Temple, USA

**Keywords:** spontaneous coronary dissection, fibromuscular dysplasia, mechanical shear forces, intramural hematoma, inflammation, endothelial repair

## Abstract

Spontaneous coronary dissection is an uncommon disorder, lacking convincing pathophysiologic evidence. Scientific statements and state-of-the-art articles suggest intramural hematoma from bleeding vasa vasorum is the cause. Evidence is based on limited invasive evaluation with optical coherence tomography. This assumption, therefore, suggests anti-coagulation be discontinued. Mechanical shear forces, intraluminal pressures do not support bleeding vasa vasorum closing a higher luminal pressure vessel.

The endothelium’s role in inflammation, thrombosis, and repair suggests the pathophysiology is failure to repair endothelium with the lack of repair as the nidus of disruption. A tear ensues and can spontaneously reseal. The lack of inflammatory cells in pathological specimens and association with another poorly understood disease fibromuscular dysplasia supports the etiology of both entities as failure to replace endothelium. The endothelium is the fulcrum of both inflammation and thrombosis. The ability to heal the rift supports conservative therapy.

Anticoagulants and antiplatelet reduce thrombosis and inflammation which will ensue when the endothelium is disrupted. These agents will substitute for the failed endothelium allowing thrombosis to be kept in check, reduce inflammation, and promote healing. This thesis and the state-of-the-art articles do not present clinical outcome data. Both support conservative interventions. Anticoagulation recommendations are however in opposite realms. Failure to repair endothelium suggest additional therapies of statins, exercise, smoking cessation will increase circulating stem cells may reduce future events and slow the progression of fibromuscular dysplasia. Future directions in understanding this disease and new therapies requires measurement of repair mechanisms such as the quantity of circulating endothelial progenitor cells.

## Introduction and background

Spontaneous coronary artery dissection is an unusual cause of myocardial infarction occurring in young women, frequently peripartum, and is associated with another unusual condition of fibromuscular dysplasia. Lipid-filled atherosclerotic plaque and inflammation of the vessel wall do not appear to be culprits. The state-of-the-art article published in the *Journal of the American College of Cardiology* describes this difficult to study disease [1]. The pathophysiology of this condition supported by this article is intramural hematoma from bleeding vasa vasorum, the “outside-in” hypothesis, and thus anticoagulation is to be avoided. This is stated in the paragraphs below along with the references cited from prior articles supporting the discontinuation of anticoagulation. The evidence for this pathophysiology is scant and suspect.

Hayes et al. [1]: “Although early inpatient anticoagulation may provide benefit by reducing thrombus burden, there are also theoretical concerns of accentuating bleeding into the IMH (intramural hematoma) leading to extension of the dissection. The general approach is to discontinue systemic anticoagulation and glycoprotein IIb-IIIa inhibitors once SCAD (spontaneous coronary artery dissection) is diagnosed unless there is apparent intraluminal thrombus or other indications for systemic anticoagulation [2].”

Hayes et al. [2]: “Because the pathophysiology, mechanisms of ischemia, PCI (percutaneous intervention) outcomes, and residua of SCAD are distinct from those associated with atherosclerotic ACS (acute coronary ischemia), many investigators have questioned the rationale and potential risks of using standard ACS therapies in patients with SCAD. For instance, early heparin use may provide benefit by reducing thrombus burden, but there are theoretical concerns about its use in the setting of acute SCAD presentation related to accentuating the risk of bleeding into the IMH or extension of dissection. Therefore, if systemic anticoagulation is started at hospital presentation, in the absence of other indications for systemic anticoagulation, consideration of discontinuation is appropriate once SCAD is diagnosed [3]."

Yip and Saw [3]: “The role of anticoagulation for SCAD is controversial with the risk of dissection extension balanced by the potential benefit of resolving overlying thrombus and improving true lumen patency. Heparin agents are typically administered for ACS patients on hospital presentation; however, we would discontinue heparin once SCAD is proven on angiography to avoid extension of IMH.”

The problem: pathophysiology is not supported

Mechanical forces do not support the "outside In" hypothesis. This conclusion is investigated by presenting the anatomy, mechanical forces on the vessel wall, and hemodynamic flow. An alternative pathophysiology is presented in the context of vessel biology. Failure to repair endothelium is the initial event and explains fibromuscular dysplasia. Failure to repair supports the anticoagulation strategy.

Anatomy

The vasa vasorum is the vascular supply of large arteries and veins greater than 0.5 mm. There are three varieties: two are arterial sources - internal and external - and one is venous. The internal travel through the vessel wall to the adventitia where they provide molecular and cellular support to the metabolism of the vessel wall. The vasa vasorum vessels cannot run in the muscular portion of the arteries due to the compressive forces of the coronary artery lumen. They do not exist in this portion of the artery other than as a transverse conduit to the adventitia. Along with these vessels, autonomic nerve fibers and lymphatics and a host of inflammatory cells are present variably based on disease and atherosclerotic burden. The adventitia is the area that is responsible for the underlying cause of myocardial infarction and will be proposed as the inflammatory brewing pot for spontaneous dissection and failure to repair. The cellular and molecular milieu regulates the inflammatory state of the vessel. This milieu is responsible for the repair of vessels and is a dynamic environment. The atherosclerotic plaque is generated when the repair is incompletely replaced by lipid deposition, fibrosis, inflammatory cells, and neovascularization [4,5].

Mechanical forces

**Figure 1:**
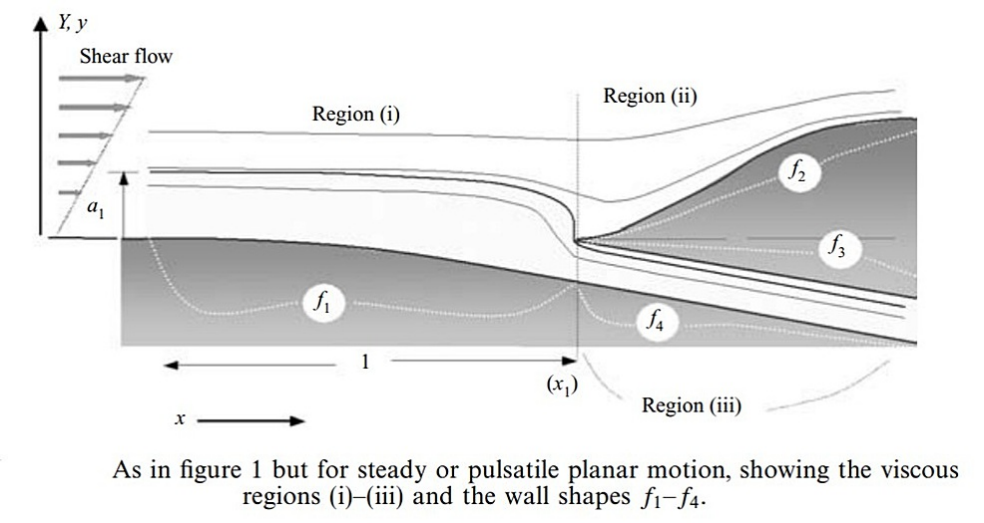
Flow Dynamics Main Channel to Side Branch Permission to reproduce from Smith et al. [7].

**Figure 2:**
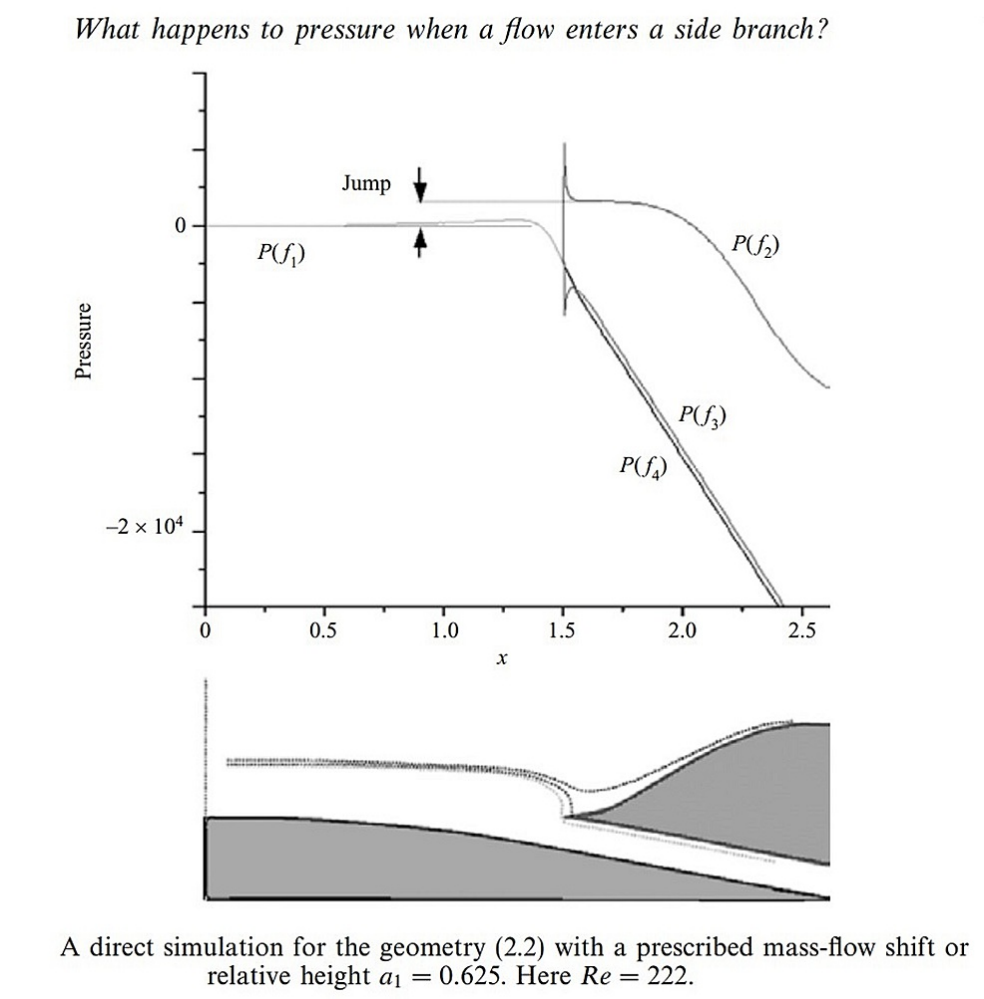
Pressure Jump in the Main Channel. Permission to reproduce from  Smith et al. [7].

The pressure of the coronary artery is always greater than the pressure in the internal vasa vasorum since the driving pressure is from the lumen of the coronary artery or after multiple branches of a systemic artery. The pressure drop is determined by the lumen size and the branching angle of the vasa vasorum. The elasticity and stiffness of the endothelium contribute. The pressure drop is greater for a small lumen oversimplified by Gorlin’s formula relating pressure gradients to the size of an orifice. The vasa vasorum is a tree-like structure with multiple branches increasing the area of supply with a mild reduction of pressure at every branch [6]. The pressure is always lower than the coronary driving pressure. The pressure of the external vasa vasorum is significantly reduced from its driving pressure of arterial supply derived from the systemic circulation. The pressure drop is significant having undergone multiple reductions in size and multiple branching. The pressure drop in the side branches has been investigated by Smith et al. [7] with the hemodynamics and pressure drop illustrated in Figure 1 and Figure 2.

There is a pressure jump in the main channel at the branch and the wall stress is the highest [7]. Atherosclerosis tends to form in these high-stress areas. Aged endothelium infiltrated with inflammatory cells and cytokines weaken the vessel wall allowing disruption. The small tear will allow pressure from the main channel to propagate in the sub-intima. The intima is dynamic and can reseal. C-reactive protein and platelets attract progenitor cells to heal the vessel [8].

Spontaneous hemorrhage in the vasa vasoroum cannot collapse the lumen of the coronary artery. The pressure is always lower outside of the artery. Abrupt closure and partial closure of the main artery will reduce the pressure distal allowing the growing obstruction to further extend. The intima can spontaneously reseal allowing the intramural hemorrhage to be absorbed by the vasa vasorum and lymphatics. Preventing thrombus and its accompanying inflammation and fibrosis would seem advantageous to healing. This mechanism favors the recommended conservative therapy in the treatment of spontaneous dissection allowing nature to heal the vessel rather than a mechanical stent that may further tear a vulnerable intima.

The strongest evidence for primary hemorrhage in the intima-media is from optical coherence tomography (OCT). OCT is an imaging technique with micrometer resolution. For reference, the endothelial cell is on average 13 by 25 microns. The technique has lateral and depth resolution which are independent of each other with lateral resolution dependent on moving the device and depth by changing the mirror depth [9]. The following reference of a series of acute coronary syndromes states “entry door type was detected in 10 and false lumen thrombus type in three patients” [10]. The paper does not favor intramural hematoma as the probable cause of dissection. Furthermore, the lateral resolution may not be accurate enough to find the disruption so the three cases may be false negatives. The author does not recommend OCT in spontaneous dissection with the risk of the procedure outweighing any benefit.

**Figure 3:**
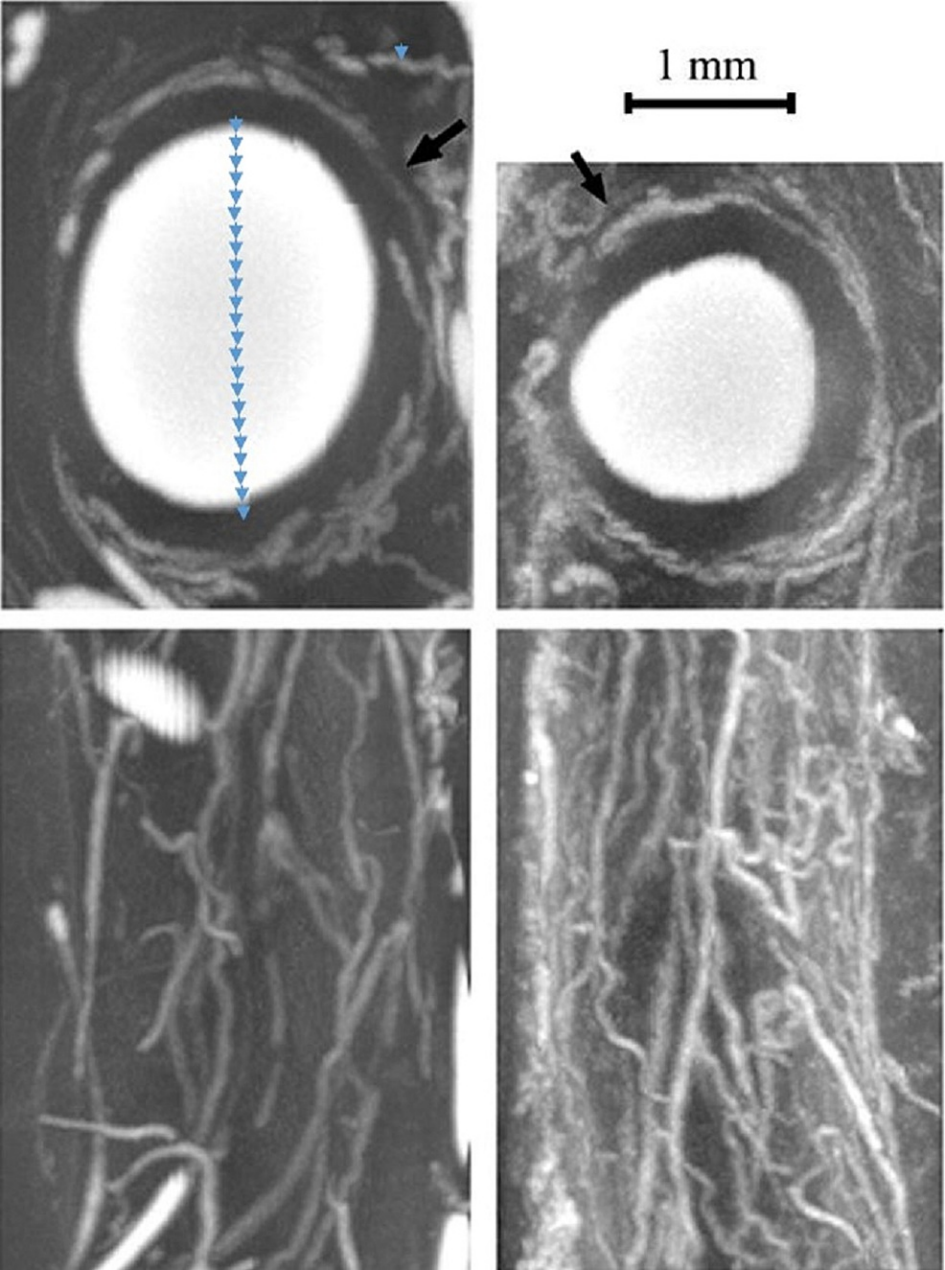
Pig Artery and Vasa Vasorum Comparing Radius Blue calibration arrows for artery diameter measurement. Black arrows represent opacified vasa vasorum. Adapted with permission from Ritman and Lerman [4].

**Figure 4:**
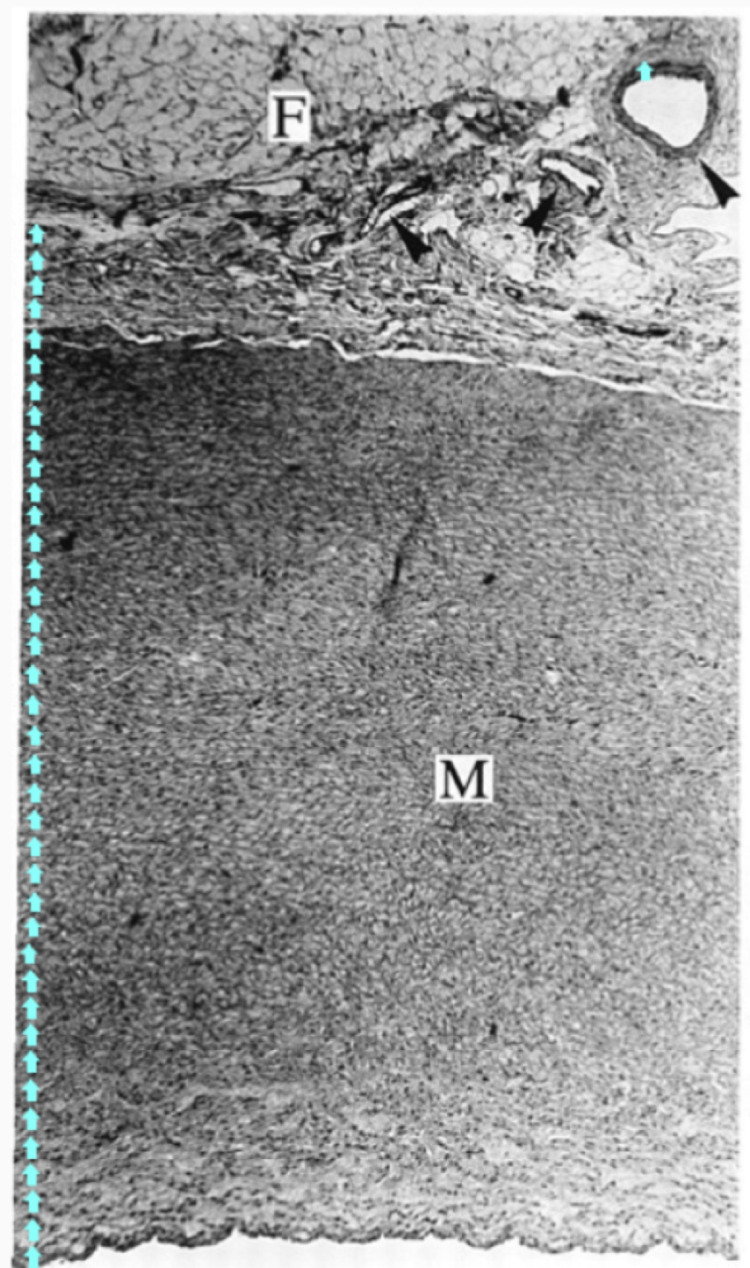
Wall Thickness of Arterial Wall and Vasa Vasorum. The black arrow is pointing to the wall of vasa vasorum; the blue arrows are calibration thickness measurement of the wall of vasa vasorum compared to the wall of the artery. M: Media; F: Periaortic Fat Adapted with permission from Stefanadis et al. [11].

Pressure x Radius / Wall Thickness determines the circumferential stress in the vessel wall. Coronary arteries are much larger in radius than the vasa vasorum by a factor of 23 (estimated from Figure 3). The vasa vasorum is smaller with a thinner wall by a factor of 36, again estimated from Figure 3 and Figure 4 [11]. The shear stress will be greater in the coronary for any pressure drop greater than 1.5 for this geometry. The geometry is illustrated by Ritman in Figure 3 of a pig coronary with injection antegrade into the arterial lumen and retrograde into the accompanied vein. The epicardial vessels have greater shear stress than the vasa vasorum. The arterial vasa vasorum punches a hole in the cylindrical artery creating point stresses that weaken and stiffen endothelium. The stress is illustrated by a stress diagram of holes placed into a thick-walled tube and thin-walled tube [12].

In addition, the vasa vasorum travels at a distance from the lumen since the compressive effects of the artery will not allow flow near the lumen. Therefore, hemorrhage is unlikely to compress the artery. This same pressure is likely to tamponade bleeding from an inflammatory disruption of the vasa vasorum. Vasa vasorum and neovascularization are more common in diseased vessels associated with atherosclerotic plaques. The young women who present with unfortunate dissections have little atherosclerosis and less vasa vasorum. Circumferential stress favors arterial intima disruption over the disruption of the vasa vasorum.

**Figure 5:**
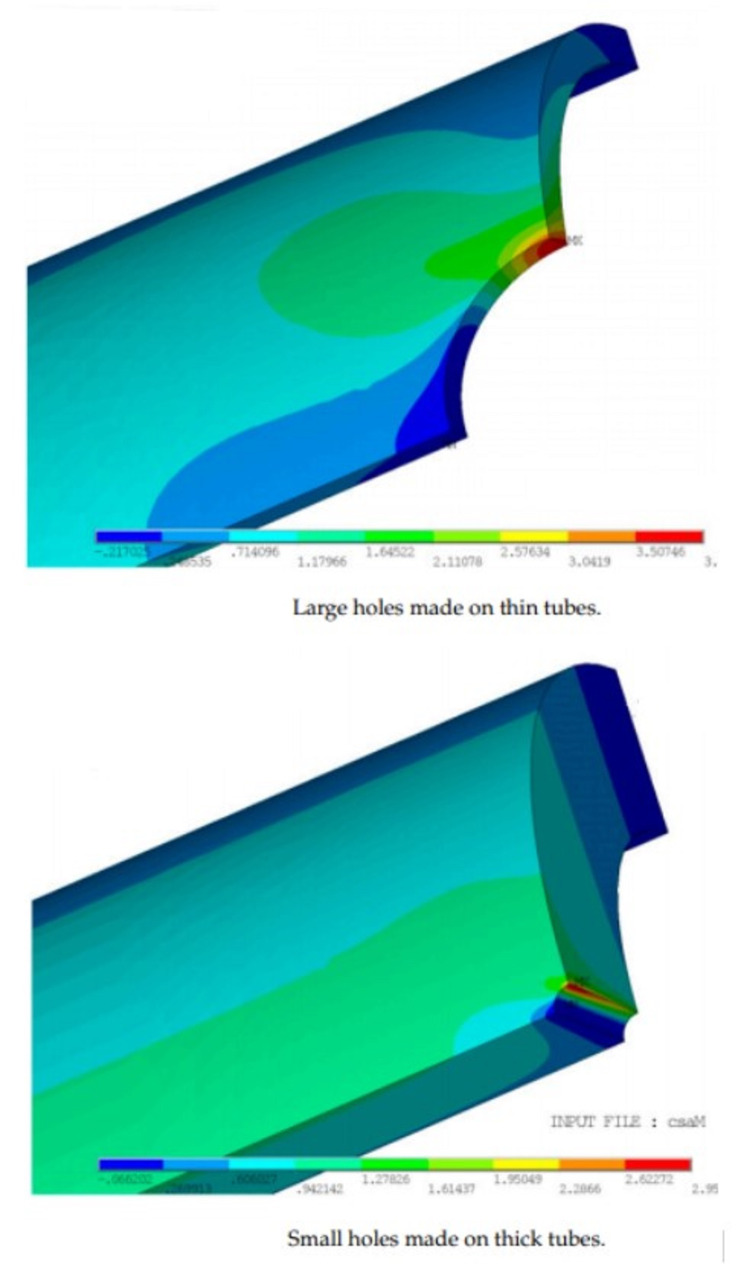
Wall Stress of Thick-Walled and Thin-Walled Tube with a Hole from a Side Branch. Adapted with permission from  Rebora and Vernassa [12].

The vasa vasorum can still be implicated in spontaneous dissection. The orifice of the vasa vasorum is an area of increased shear (Figure 5). Tears are more likely to occur in areas of increased shear. Additional factors of endothelial denuding are seen in plaque erosion, frequently seen in young female smokers. Endothelial denuding can occur in a normal vessel when an aged endothelial cell is not replaced or when localized inflammation destroys the endothelial lining. The inflamed intima now exposed precipitates coronary media contraction with resultant dissection flap and entry of intraluminal blood into the media. Healing of the endothelium from circulating progenitor cells resolves the problem, occurring frequently within 24-48 hours [13,14]. The rapid healing supports conservative therapy.

Anticoagulation has been successful in acute coronary syndromes. In the early days of angioplasty, plaque dissection is considered a success. Anti-coagulation prevented thrombosis and did not cause further bleeding into the vessel wall. The media and adventitia actively diffuse solute and proteins to keep the vessel healthy. A thrombus formation would significantly decrease this function.

## Review

Thrombosis inflammation repair

Biology is efficient and complex, not simple. Pathophysiology of thrombosis in a coronary artery is an example of one-dimensional thinking delaying the development of new therapies for coronary artery disease. Half a century of one-dimensional thinking has been directed to treating thrombosis. From the first visualization of thrombus in a coronary, a war has been raged to develop fibrinolytics, anticoagulants, and antiplatelets. These new therapies have improved outcomes in myocardial infarction. Including two-dimensional thinking of clotting and inflammation gives a clearer picture of the causes of thrombosis. JUPITER (Justification for the Use of Statins in Primary Prevention: An Intervention Trial Evaluating Rosuvastatin trial) [15], CANTOS (Canakinumab Anti Inflammatory Thrombosis Outcomes Study) [16], CHOLCOT (Colchicine Cardiovascular Outcomes Trial) [17] are finally opening the door to the cause of thrombosis with the promise of anti-inflammatory therapies. Three-dimensional thinking will also include repair mechanisms.

Inflammation is a term used frequently without understanding what it represents. The paper “'Observational Medicine' Should be Replaced by 'Real Science'” proposed fundamental laws of biology. Law 5 addressed inflammation: “There must be a distinction between self and the environment. (Immunity and inflammation are the defenses against invaders from the environment and responsible for repair of damaged and senile cells.)” [18]. Inflammation has two characteristics that appear to be opposed - killing and rejuvenation. Law 5 is like a mousetrap ready to spring into a cytokine storm. The reaction must be balanced so the storm can be stopped, shifting from killing to repair of damage.

Inflammation and thrombosis are intertwined. Inflammation and clotting are both balanced and ready to leap between destruction of invaders and repair, or thrombosing a bleeding vessel and removing the clot allowing free flow of blood elements. The clotting cascade must be stopped and reversed to prevent the entire vasculature from turning into a large clot. Both processes are like a teeter-totter demonstrating balance during health. The endothelium is the moderator “fulcrum.” The endothelium has properties that control both thrombosis and inflammation. Forgotten endothelium exists in lymphatic vessels one of the major components of the immune system and runs with the vasa vasorum. This system also participates and is affected by both inflammation and thrombosis. The transport of inflammatory cells, repair cells, and proteins by the lymphatics contributes to the cause of coronary dissection. This statement deserves further explanation!

The endothelium is remarkable being able to contact flowing blood without causing clotting. One of the mechanisms is surface expression of heparan sulfate proteoglycans. These natural anti-coagulants also have a dual role also initiating inflammatory cascade. Exogenous heparins have a mixture of proteoglycans and is also a moderator of inflammation by inhibiting the function of neutrophils, preventing expressions of inflammatory mediators, inhibiting proliferation of vascular smooth muscle cells, and by its anticoagulant activity reducing thrombin generation, a potent stimulator of the immune response [19]. In dissection, the endothelium is disrupted outside-in, or the basement membrane is exposed inside-out. Both scenarios would benefit from the anti-inflammatory and anticoagulant effects of heparin.

The inflammatory cascade and thrombosis are also mediated by platelets. Platelets represent a link between the hemostatic system and immune system. Activated platelets release a myriad of proteins which is remarkable since these small cells do not have a nucleus. These proteins cause pro thrombosis, activate leucocyte, enhancing inflammatory mediators and enhance healing by recruiting circulating progenitor cells [20,21]. The tiny platelet is truly three-dimensionally involved in thrombosis, inflammation, and healing. Anti-platelet medications have been shown to decrease inflammation with the most evidence related to C-reactive protein (CRP).

CRP is a protein existing in two forms: pentameric and monomeric. CRP protein production is in the liver predominantly and to a lesser extent in endothelial cell and smooth muscle cells. It is a marker of inflammation and is elevated in infection, immunization, and in cancer. As a biomarker, it reflects the activity of the immune system. Hs-CRP (high-sensitivity C-reactive protein) levels greater than 2 predict future cardiovascular events. This criterion is selected in trials lowering CRP with reduction of cardiovascular events. Lifestyle changes also reduce CRP and reflect traditional risk factors for cardiovascular disease. CRP is found in atherosclerotic plaque. It shifts Th1/Th2 balance toward Th1 favoring vascular injury [22].

The blood vessel inflammatory, thrombosis, and repair introduction is necessary to propose a mechanism for coronary artery dissection. Intramural hematoma or external tear are the two proposed mechanisms, a one-dimensional view. A more complete analysis would also include why was there intramural bleeding and what preceded the tear which resulted in dissection? Eosinophils are found; however, there is a lack of macrophage and lymphocytic infiltration in pathological specimens implying inflammation is not causal.

**Figure 6:**
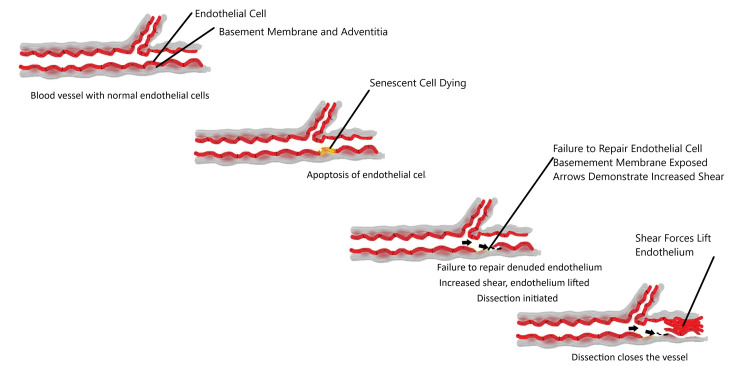
Failure to Repair Endothelium, a Nidus for Increasing Shear "Arrows" Causes a Dissection Allowing Blood Flow to Separate Endothelial Lining From the Media. Sequence: top to bottom - normal vessel; senescent cell not replaced; endothelium-denuded; increase shear due to absent endothelium; and shear forces dissect the endothelium from the vessel wall.

Failure to replace senile endothelium is one mechanism. Endothelial cells live between 45 days (about one and a half months) and 60 years. Replacing senile endothelial cells is the job of the immune system and circulating progenitor cells. The lifespan of an endothelial cell depends on the shear induced, greatest at areas of bifurcation. The high shear areas are ripe for atherosclerosis. The life span of the cells lining vessels, therefore, depends on the location. Endothelial cells can be detached by inflammatory response or by senescence. Once the lining is disrupted, dissection can ensue (Figure 6). The disruption can occur in the vasa vasorum or in the coronary artery since they are both lined with endothelium. The forgotten lymphatics transport immune cells and progenitor cells. They fulfill law 5, defending against invaders, by transporting immune cells and transport progenitor cells to repair damage previously caused by the immune cells.

**Figure 7:**
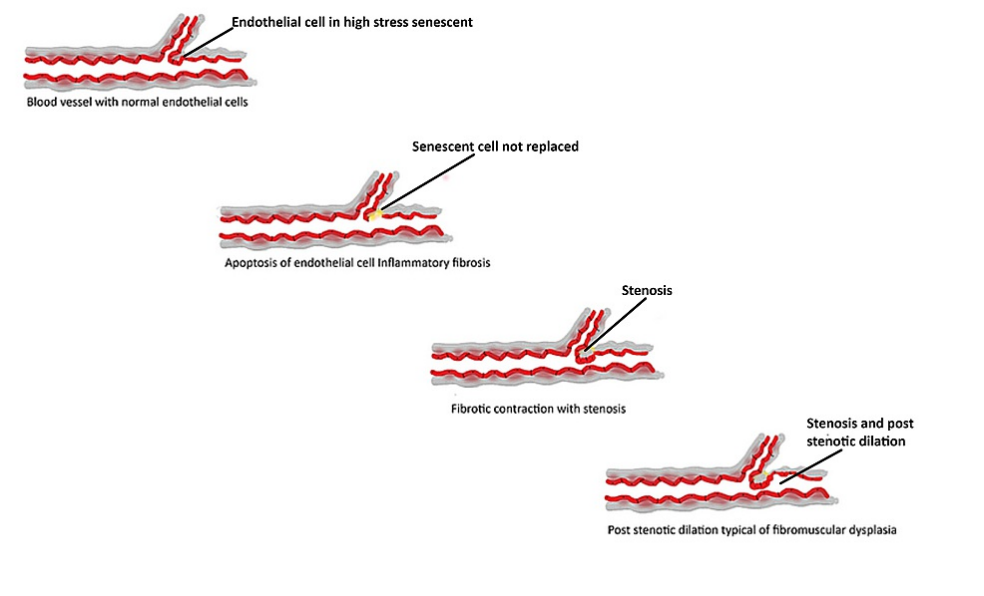
Failure to Repair Endothelium With Fibrotic Healing Causes Fibromuscular Dysplasia. Sequence: top to bottom - aging endothelial cell; not replaced with incomplete fibrotic healing; contracture with stenosis; and typical bead of pearls with post stenotic dilation.

It is suggested that 40 to 90 percent of dissection patients who are screened with computed tomographic angiography demonstrate areas of fibromuscular dysplasia [23,24]. The pathophysiology of this condition is unknown demonstrating beaded stenosis with fibrosis of the intima, media, and adventitia with dilation distal to the stenosis. The etiology of dissection and fibromuscular dysplasia must be linked. The author proposes inadequate repair mechanisms as a pathologic mechanism for both dissection and fibromuscular dysplasia. Failure to replace senile or damaged endothelium will initiate an incomplete post-natal repair with fibrocytes resulting in scarring and stenosis of the vessel. Dilation distal to the stenosis is a flow mediated process (Figure 7). A risk factor for inadequate repair is smoking. Smoking reduces circulating stem cells. Estrogen (hormonal risk) promotes mobilization of stem cells when studied in mice given fixed doses of estrogen. Deficit of estrogen reduces circulating stem cells. Estrogen is a complex hormone that influences the immune system and does so by changing its concentration monthly during menses and pregnancy. The transient deficit of estrogen will influence repair and inflammatory response explaining female predisposition. The complex interactions of estrogen and the immune system was investigated in the a paper [25]. The article explains the paradoxical role of estrogen in cardiovascular health. The effort in this paper relates immune modulation by hormones.

The above pathophysiology allows for therapeutic recommendations of smoking cessation, exercise, statin medications which will increase circulating stem cells promoting healing of endothelium. Heparin anticoagulants and antiplatelet medication decrease the inflammatory response caused by dissection and promotes healing of the disrupted endothelium.

Assessment of models of dissection

Intramural Hematoma versus Inflammatory Disruption or Failure to Repair Endothelium

Table 1 compares pathophysiology of failure to repair and intramural hematoma. The table suggests failure to repair is a better model for the unusual condition of coronary dissection.

**Table 1:** Comparison of Pathophysiology Failure to Repair and Intramural Hematoma. FMD: fibromuscular dysplasia.

	Failure to Repair	Bleeding Vasa Vasorum Intramural Hematoma
Explains initiating event	Yes denudation of endothelium	No
Explains association with FMD	Yes incomplete healing after denudation with fibrosis	No
Female predominance	Yes reduced circulating stem cells estrogen associated [26]	No
Supports conservative approach	Yes	Yes
Supports anticoagulation	Yes	No
Mechanical forces support vessel closure	Yes	No

Bleeding into the vasa vasorum is unlikely to close the vessel. The growth of the hematoma depends on the driving force pressure of the vasa vasorum which is always lower than the coronary lumen. The vasa vasorum and lymphatic vessels running within the artery wall are causal in atherosclerosis promoting the appearance of inflammatory cells, plaque rupture, and dissection. They are causal by failing to deliver cell replacement for damaged endothelium. The processes may have different endpoints; however, the milieu is the same. The vasa vasorum and lymphatics are leaky providing nourishment to the cells of the artery. Lipids, immune cells, antibodies, clotting factors, are delivered to the wall and can infiltrate the vessel wall forming plaque, neovascularization of the plaque, and inflame the plaque making it vulnerable. Failure to replace aged endothelium, the fulcrum of both inflammation and thrombosis is the catalyst for adverse cardiovascular events. Reducing inflammation with heparin, antiplatelets statins, colchicine is a successful strategy for coronary syndromes. Dissection is part of this spectrum. Dissection exposes the muscularis to blood stimulating spasm of the muscularis. Dissection patients will have recurrent pain due to spasm until the anti-inflammatory effects of heparin and antiplatelets rebalances the immune system’s exaggerated and deleterious response.

In cases of multivessel disease, anti-inflammatory therapy may need to be escalated as described in the referenced articles [27-29]. Plasmapheresis, intravenous immune globulin, and steroids were necessary to reverse the fetal antigen-mediated multivessel immune-mediated coronary syndrome.

## Conclusions

The pathophysiology of failure to replace endothelium is consistent with the observations in spontaneous coronary dissection. These observations include lack of inflammatory cells, reduced number of vasa vasorum, the association with fibromuscular dysplasia, female sex predominance, association with pregnancy, and reduction in estrogen levels. The increased shear by denuded endothelium supports mechanical disruption that initiates the dissection flap. Anticoagulation and antiplatelet are necessary to counter the prothrombotic and inflammatory consequences of denuded endothelium.

The current literature supports bleeding vasa vasorum as the etiology of intramural hematoma and theoretically recommends cessation of anticoagulant and antiplatelets. This pathophysiology fails to explain the initiating event, the association of fibromuscular dysplasia, female predominance, and reduction in vasa vasorum. Mechanical forces do not favor intramural hematoma as the pathophysiology of spontaneous dissection. OCT evidence is mixed and may not have the resolution to exclude an entry tear. There are no clinical outcomes to support either opinion so the readers must select a strategy.

There is no therapy to predict or prevent bleeding vasa vasorum. Measurement of circulating progenitor cells could predict patients at risk. Therapies to increase progenitor cells such as exercise, statin therapy, and smoking cessation may impact recurrence of dissection and fibromuscular dysplasia. Anticoagulation supports the repair of the injured endothelium.

The state-of-the-art papers and the above thesis support conservative interventions. Therefore, anticoagulation recommendations are in opposite realms based on the pathophysiology of failure to repair versus intramural hematoma.

Future perspectives

A new target for therapy beyond thrombosis and inflammation is failure to repair. Routine measurement of circulating progenitor cells will enlighten degenerative disease processes with no known pathophysiology. This is the experiment to prove dissection is a failure of repair due to a transient lack of progenitor cells correlated to hormonal control. New therapeutics should be tested for their effect on circulating stem cells and the inflammatory system. Coronary dissection and fibromuscular dysplasia are uncommon examples of failure to repair. More common examples of failure to repair are age, degenerative arthritis, dementia, chronic kidney disease, and most chronic disease states. These conditions have reduced circulating progenitor cells. Therapeutics directed at increasing these repair mechanisms should be a priority of future investigations. 
